# Global Longitudinal Strain at Rest as an Independent Predictor of Mortality in Liver Transplant Candidates: A Retrospective Clinical Study

**DOI:** 10.3390/jcm9082616

**Published:** 2020-08-12

**Authors:** Mare Mechelinck, Bianca Hartmann, Sandra Hamada, Michael Becker, Anne Andert, Tom Florian Ulmer, Ulf Peter Neumann, Theresa Hildegard Wirtz, Alexander Koch, Christian Trautwein, Anna Bettina Roehl, Rolf Rossaint, Marc Hein

**Affiliations:** 1 Department of Anesthesiology, Faculty of Medicine, RWTH Aachen University, 52074 Aachen, Germany; bianca.hartmann@rwth-aachen.de (B.H.); aroehl@ukaachen.de (A.B.R.); rrossaint@ukaachen.de (R.R.); mhein@ukaachen.de (M.H.); 2 Department of Internal Medicine I, Cardiology, Angiology and Internal Intensive Care Medicine, Faculty of Medicine, RWTH Aachen University, 52074 Aachen, Germany; s.hamada@gmx.de; 3 Clinic for Cardiology, Nephrology and Internal Intensive Care, Rhein-Maas Klinikum, 52146 Würselen, Germany; Michael.Becker@rheinmaasklinikum.de; 4 Department of General, Visceral and Transplantation Surgery, Faculty of Medicine, RWTH Aachen University, 52074 Aachen, Germany; aandert@ukaachen.de (A.A.); fulmer@ukaachen.de (T.F.U.); uneumann@ukaachen.de (U.P.N.); 5 Department of Internal Medicine III, Gastroenterology, Metabolic Diseases and Intensive Care, Faculty of Medicine, RWTH Aachen University, 52074 Aachen, Germany; thwirtz@ukaachen.de (T.H.W.); akoch@ukaachen.de (A.K.); ctrautwein@ukaachen.de (C.T.)

**Keywords:** strain analysis, echocardiography, cirrhotic cardiomyopathy, cardiac systolic function, dobutamine stress test, outcome

## Abstract

Speckle tracking echocardiography enables the detection of subclinical left ventricular dysfunction at rest in many heart diseases and potentially in severe liver diseases. It could also possibly serve as a predictor for survival. In this study, 117 patients evaluated for liver transplantation in a single center between May 2010 and April 2016 with normal left ventricular ejection fraction were included according to clinical characteristics of their liver disease: (1) compensated (*n* = 29), (2) clinically significant portal hypertension (*n* = 49), and (3) decompensated (*n* = 39). Standard echocardiography and speckle tracking echocardiography were performed at rest and during dobutamine stress. Follow-up amounted to three years to evaluate survival and major cardiac events. Altogether 67% (78/117) of the patients were transplanted and 32% (31/96 patients) died during the three-year follow-up period. Global longitudinal strain (GLS) at rest was significantly increased (became more negative) with the severity of liver disease (*p* < 0.001), but reached comparable values in all groups during peak stress. Low (less negative) GLS values at rest (male: >−17/female: >−18%) could predict patient survival in a multivariate Cox regression analysis (*p* = 0.002). GLS proved valuable in identifying transplant candidates with latent systolic dysfunction.

## 1. Introduction

For liver transplant recipients, heart diseases are an important risk factor for perioperative mortality [1]. Approximately 7–21% of deaths following liver transplantation are related to cardiovascular events [2]. Ischemic events or acute heart failure may be associated with concomitant coronary artery disease, valvular disease, or cardiomyopathy. A special form leading to systolic dysfunction is cirrhotic cardiomyopathy (CCM), which has been related to liver cirrhosis and portal hypertension [3]. In patients with CCM, 17% of death in the perioperative phase are attributable to cardiac causes [4]. The main triggers of CCM are hemodynamic stress (high cardiac output as a consequence of peripheral vasodilation) and inflammation [5]. These triggers lead to progressive disease starting with diastolic dysfunction, followed by an increasing reduction of the contractile reserve and finally impaired systolic function at rest [6]. In most patients, the working capacity is so severely restricted by liver insufficiency that impaired cardiac function is not detectable by evaluations of working capacity. Thus, an accurate assessment of left ventricular (LV) function in patients awaiting liver transplantation is crucial for risk stratification [7].

No accepted gold standard for the diagnosis of CCM exists [8], but echocardiography is by far the most preferred method of diagnosis [9]. Of the echocardiographic parameters, LV ejection fraction (LVEF) in particular is widely used to quantify LV systolic function [10,11]. In 2005, the World Congress of Gastroenterology proposed a resting LVEF below 55% or a reduced increase in cardiac output in any stress test as the definition of systolic dysfunction in cirrhosis [12]. However, there are numerous publications showing that LVEF cannot adequately map LV systolic function, especially in the early phase of systolic dysfunction [11,13,14]. Myocardial function can fluctuate significantly without any changes in LVEF [14]. Thus, LVEF is not a good parameter for contractility [8], as it is also influenced by preload, afterload and heart rate [15,16]. Even the prognostic value of LVEF is still controversial [17,18]. A better predictor of mortality in acute heart failure seems to be global longitudinal strain (GLS) [18].

Strain analysis is mostly performed by 2D speckle tracking echocardiography: small areas in a region of interest are identified and tracked over the cardiac cycle to assess myocardial deformation [19]. The usefulness of this method has already been demonstrated in numerous clinical settings [19]. In particular, GLS is often used and considered to be more sensitive to subclinical ventricular dysfunction than standard echocardiography [20,21]. However, this parameter is also influenced by load [22] and a number of clinical factors, such as sex and age [23,24].

Despite these limitations, given the promising results in other clinical settings, information from echocardiographic strain analysis could possibly improve the assessment of cardiac systolic function prior to liver transplantation and prove beneficiary in predicting survival of these patients. Accordingly, the aim of this study is to assess the predictive value of strain analysis for mortality of liver transplant candidates with normal ejection fraction. Therefore, strain analysis was performed by 2D speckle tracking both at rest and during a dobutamine stress echocardiography (DSE).

## 2. Material and Methods

### 2.1. Patient Cohort

**Figure 1:**
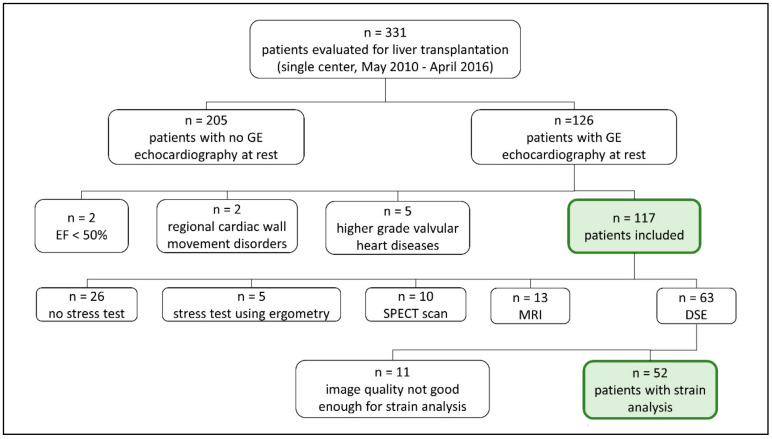
Overview of the inclusion and exclusion criteria.

This study was approved by the local ethics committee of the Uniklinik RWTH Aachen (EK 291/13; date of approval 22 June 2016) and conducted in accordance with the Declaration of Helsinki. According to the ethics vote, informed consent of the patients was not required. All patients evaluated for liver transplantation in an inpatient setting between May 2010 and April 2016 at the Uniklinik RWTH Aachen (Aachen, Germany), with a minimum age of 18 years at the time of examination, were screened for retrospective study enrollment. Patients with an echocardiographic examination at rest performed using a GE ultrasound device (GE Healthcare, Milwaukee, WN, USA) recorded on the server and without regional wall movement disorders or high-grade heart valve disease (>I°) and a normal LVEF at rest (≥50%) were subsequently included in the study. Figure 1 provides an overview of all inclusion and exclusion criteria.

In order to assess the influence of the stages of liver disease as a possible trigger of CCM, the patient cohort was divided into 3 groups according to the classification described by de Franchis et al. [25] based on the status at the time of examination: (1) patients with compensated advanced liver disease (cALD); (2) patients with cALD and clinically significant portal hypertension (CSPH); and (3) patients with decompensated advanced liver disease (dALD). Clinically significant portal hypertension was defined as proven gastroesophageal varices or a platelet count below 150,000 [25]. The definition of decompensation for dALD was based on the presence of one of the following features: hydropic decompensation, transjugular intrahepatic portosystemic shunt, hepatorenal syndrome, or spontaneous bacterial peritonitis. Hepatorenal syndrome and spontaneous bacterial peritonitis were diagnosed according to Lerschmacher et al. [26].

### 2.2. Patient Characteristics

Detailed information, such as baseline demographics, systolic blood pressure (sBP) and diastolic blood pressure (dBP), heart rate, blood values, cardiovascular risk factors and medications, was obtained from the records. In detail, these were the following data collected for each individual patient: sex, age, presence of diabetes mellitus or arterial hypertension, intake of antihypertensive or diuretic drugs, and blood values of creatinine, bilirubin, international normalized ratio, platelets, C-reactive protein, sodium, hemoglobin, and albumin. The Model for End-Stage Liver Disease (MELD) score was calculated to assess the individual clinical status. All patients included were monitored for 3 years with regard to complications such as major cardiac events (MACE), transplantation, and survival, if possible. MACE was defined as one of the following: death, heart failure (both left and right), and ischemic cardiac events.

### 2.3. Echocardiography at Rest

All echocardiographic examinations at rest were performed with a GE Vivid 7 ultrasound system (General Electric (GE) Healthcare, Buckinghamshire, GB) combined with a 5S ultrasound probe (GE) at a frame rate of 56–92 s^−1^. Apical 2-, 3-, and 4-chamber views, parasternal long-axis, and parasternal short-axis images at 3 different levels (mitral valve, papillary muscles and apical) were saved as 2-dimensional greyscale cine-loops for subsequent analysis.

LVEF and LV volumes were determined from the apical 2- and 4-chamber views using the biplane Simpson method. According to Agricola et al. [27], an increase in LVEF during DSE of less than 5% was considered as limited contractile reserve. Tricuspid annular plane systolic excursion (TAPSE) was assessed in M-mode and mitral annulus early diastolic velocity (E’) was identified by tissue Doppler imaging in a 4-chamber view. Mitral inflow velocities, such as transmitral peak early passive filling velocity (E), late diastolic filling velocity caused by atrial contraction (A) and deceleration time, were determined using PW-Doppler over the mitral valve in 4-chamber views. The degree of diastolic dysfunction (°I–°III) of each patient was assessed according to the criteria of Nagueh et al. [21].

Analyses of the two-dimensional speckle tracking echocardiography recordings at rest (both long- and short-axis images) were acquired with EchoPac-PC (Version 112, GE Healthcare, Buckinghamshire, GB) by a single examiner. The region of interest was manually placed along the endocardium (while excluding the papillary muscle), and the width was adjusted to the wall thickness. The region of interest tracked by the computer over the cardiac cycle was visually checked and manually adjusted as needed. All measured parameters were averaged from several consecutive beats. Global values for longitudinal (GLS), radial, and circumferential strain were calculated from the appropriate views [28,29].

Abnormal GLS values were defined according to the publication by Asch et al. using the values at rest without pausing beta blockers [30]: values below −26 for females and −24 for males were defined as high, and values above −18 and −17 were defined as low. Thus, more negative values are termed higher. This resulted in 3 categories: patients with (1) low GLS, (2) normal GLS, and (3) high GLS. For the multivariate analysis, a GLS increase during DSE of <2% was defined as pathological according to Lancellotti et al. [31].

To calculate the LV twist, apical heart rotation (i.e., circumferential strain) was subtracted from the basal rotation as described by Mielczarek et al. [32].

### 2.4. Stress Echocardiography

All patients were instructed to pause beta blockers, calcium antagonists and nitrates for 72 h before DSE. The Philips iE33 ultrasound device was used with an X5-1 probe. DSE was started with a dobutamine flow rate of 10 µg/kg BW/min. Subsequently, the flow rate was increased every 3 min by 10 µg/kg BW/min up to 40 µg/kg/min to reach an age-adapted target heart rate [33]:

maximum heart rate = (220 − age) × 0.85.(1)

Images were evaluated at 4 defined time points: before (base), at low dobutamine infusion dose (10 µg/kg BW/min), at maximum dobutamine infusion dose (peak), and after stopping the dobutamine infusion and recovering baseline heart rates (post). The scans were digitally stored for offline analysis with a frame rate of 50–58 s^−1^. During the examination, echocardiography, sBP, and dBP were monitored and recorded continuously. Mean BP was calculated as

mean BP = dBP + 1/3 × (sBP − dBP).(2)

Image analysis of stress echocardiographic recordings was performed using TOMTEC Imaging Systems (TOMTEC Imaging Systems, Unterschleissheim, Germany). Here, the endocardium was marked in end systole, and subsequent automated tracking was manually corrected if needed [34]. EF and stroke volume index (SVi) were calculated from left ventricular volumes. The stroke work index was obtained by multiplying the mean arterial pressure and SVi. GLS was calculated from apical views [34].

### 2.5. Statistical Analysis

Statistical evaluation was performed using SPSS IBM Statistics version 25 (IMB Corp., Armon NY, USA), and graphs were created with GraphPad Prism version 8 (GraphPad Software, San Diego, CA, USA). Continuous data are presented as the mean ± standard deviation, and categorical variables are presented as numbers and percentages. Between-group differences were investigated by means of univariate analysis of variance with Tukey’s post hoc test, chi-square test or variance analysis for repeated measurements. Contrast analysis with baseline values as a reference was used to describe the effect of time and dobutamine dosage. The effect of predefined predictors for survival (severity of liver cirrhosis, MELD, GLS, LVEF, body mass index, transplantation, age, and diastolic dysfunction) was assessed by Cox regression analyses. Significant predictors from the univariate analysis were included in a stepwise forward multivariate approach after sorting by *p*-values. *p*-values < 0.05 were considered statistically significant.

## 3. Results

### 3.1. Patient Selection and Group Allocation

Of the 331 patients screened for study inclusion (from May 2010 to April 2016), a total of 214 were excluded. The reasons were a missing echocardiography study at rest performed by a GE device in 205 cases, a reduced EF (<50%) in two cases, regional cardiac wall movement disorders in 2 cases, and high-grade valvular heart diseases in five cases (Figure 1). Thus, 117 patients were enrolled. Strain analysis both at rest and under dobutamine stress was available in 52 of these patients. No stress test was performed in 26 cases, alternative techniques (such as stress test using ergometry, single-photon emission computed tomography or magnetic resonance imaging) were used in 28 patients, and DSE image quality was too poor for strain analysis in 11 cases.

The 117 patients included in the study were divided as follows: 29 patients in the cALD group, 49 patients in the CSPH group, and 39 patients in the dALD group. Of these patients, strain analysis during DSE was performed in eight (cALD), 26 (CSPH) and 18 (dALD) patients.

### 3.2. Patient Characteristics

Baseline characteristics of patients are listed in Table 1. The three most frequent etiologies of liver disease were ethyltoxic in 31.6% (37 out of 117), viral in 17.9% (21 out of 117) and tumor-related in 12.8% (15 out of 117). Although the distribution of diagnoses was not equal in the three groups (*p* < 0.001), GLS at rest (*p* = 0.129) or LVEF (*p* = 0.532) did not differ significantly between them.

There were no significant differences among groups in age (*p* = 0.538), body mass index (*p* = 0.830), sex (*p* = 0.524), or sBP (*p* = 0.106). Patients in the dALD group had significantly higher MELD values than those in the cALD group (*p* = 0.005), higher C-reactive protein values than those in the CSPH group (*p* = 0.014), and lower hemoglobin (*p* = 0.002 resp. *p* = 0.045) and albumin values (*p* = 0.002 resp. *p* = 0.035) than those in both other groups. All patients in the CSPH and dALD groups had lower platelet counts (all *p* < 0.001) and lower diastolic blood pressure than the patients in the cALD group (*p*_Group_ = 0.009). The medication only differed significantly among groups regarding beta blockers (*p* = 0.005) and diuretics (*p* = 0.001): Patients in the cALD group took significantly fewer beta blockers (*p* = 0.002) and diuretics (*p* < 0.001).

### 3.3. Echocardiographic Findings at Rest

Parameters describing ventricular dimensions, as well as systolic and diastolic function at rest, are shown by group in Table 2. Patients in the cALD, CSPH and dALD groups had comparable ventricular dimensions. From the parameters describing left ventricular function, only GLS displayed significant differences (*p* < 0.001): CSPH and dALD patients showed higher GLS values than cALD patients. Of all patients in the cALD group, 24% (7 out of 29) had low, 62% (18 out of 29) had normal and 3% (1 out of 29) had high GLS values. In the CSPH group 8% (4 out of 49) were identified as low, 61% (30/49) as normal and 18% (9 out of 49) as high GLS values, and in the dALD group, 8% (3 out of 39) were identified as low, 62% (24 out of 39) as normal and 28% (11 out of 39) as high GLS values. TAPSE demonstrated the highest values in the dALD group (*p* < 0.05). From the diastolic parameters, E/A was significantly higher in the CSPH group and E as well as left atrial volume index were significantly higher in the CSPH and dALD groups compared to the cALD group. Thus, the degree of diastolic dysfunction was higher in patients with CSPH and dALD.

In resting echocardiography, patients with beta blockers had significantly lower GLS values in all groups than patients without beta blockers (cALD: −17 ± 2 vs. −19 ± 2; CSPH: −21 ± 3 vs. −24 ± 2; dALD: −21 ± 4 vs. −24 ± 3) (*p* < 0.001). However, this effect did not significantly differ between cALD, CSPH, and dALD (*p* = 0.693).

### 3.4. Echocardiographic Findings during DSE

**Figure 2:**
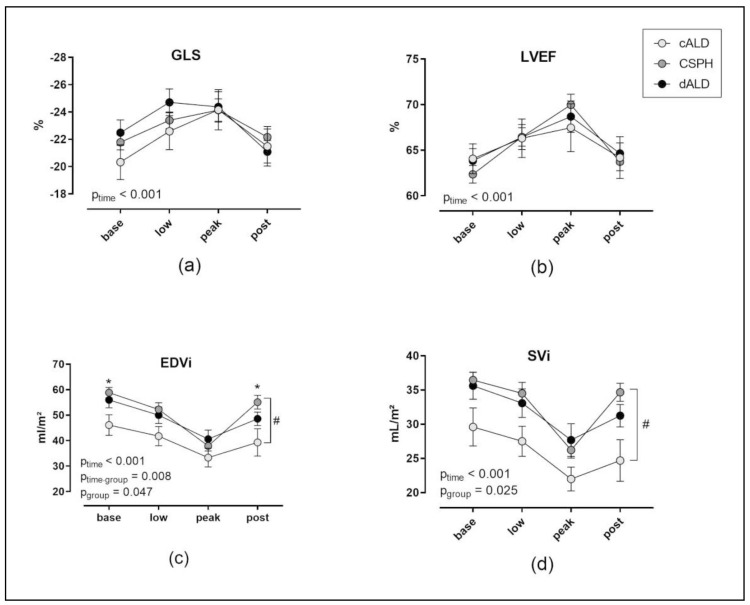
Effect of dobutamine stress echocardiography (DSE) on left ventricular function related to the severity of liver disease. The values at ‘base’ were recorded at rest before DSE, at ‘low’ with a dobutamine rate of 10 µg/kg BW/min, at ‘peak’ with the maximum dobutamine rate and at ‘post’ after stopping dobutamine and returning to baseline heart rates (mean +/− SD). The following parameters of left ventricular function are shown (**a**) global longitudinal strain (GLS), (**b**) left ventricular ejection fraction (LVEF), (**c**) end-diastolic volume index (EDVi) and (**d**) stroke volume index (SVi) (# *p* ≤ 0.05 between groups and * *p* < 0.05 vs. cALD at time point).

Heart rate at rest was significantly lower in the CSPH group than in the other two groups (*p* = 0.003 vs. cALD and *p* = 0.002 vs. dALD) and increased significantly during stress in all groups (Table 3). The predefined maximum heart rate was achieved on average in all groups. The peak heart rate did not differ significantly between the groups (*p* = 0.066), nor did the mean arterial pressure demonstrate any significant differences between the groups (*p* = 0.311). The LV end-systolic volume index and stroke work index decreased significantly without differences among groups (Table 3). GLS, LVEF and the cardiac index increased significantly during stress (*p* < 0.001) without differences between groups (Figure 2 and Table 3). DSE caused a significant reduction in the end-diastolic volume index (EDVi) and SVi (*p* < 0.001 each), with significant differences among groups (p_EDVi_ = 0.047 and p_SVi_ = 0.025).

### 3.5. Follow-Up Analysis

Within 3 years after the evaluation for possible liver transplantation, 66.7% (78/117) of the patients had received a donor liver. The waiting period for a donor organ for patients who received a transplant was 150 ± 137 (cALD group), 147 ± 164 (CSPH group), and 151 ± 152 days (dALD) from the time of evaluation. Twenty-one patients (17.9%) were lost in the three years of follow-up (five with cALD, 10 with CSPH, and six with dALD). These were mainly patients without transplantation, as the others were regularly seen for aftercare. Of the patients who were followed up for three years, 32.3% (31 out of 96) died during the study period. The main cause of death was multiple organ failure from sepsis in 54.8% (17 out of 31) of all groups.

**Figure 3:**
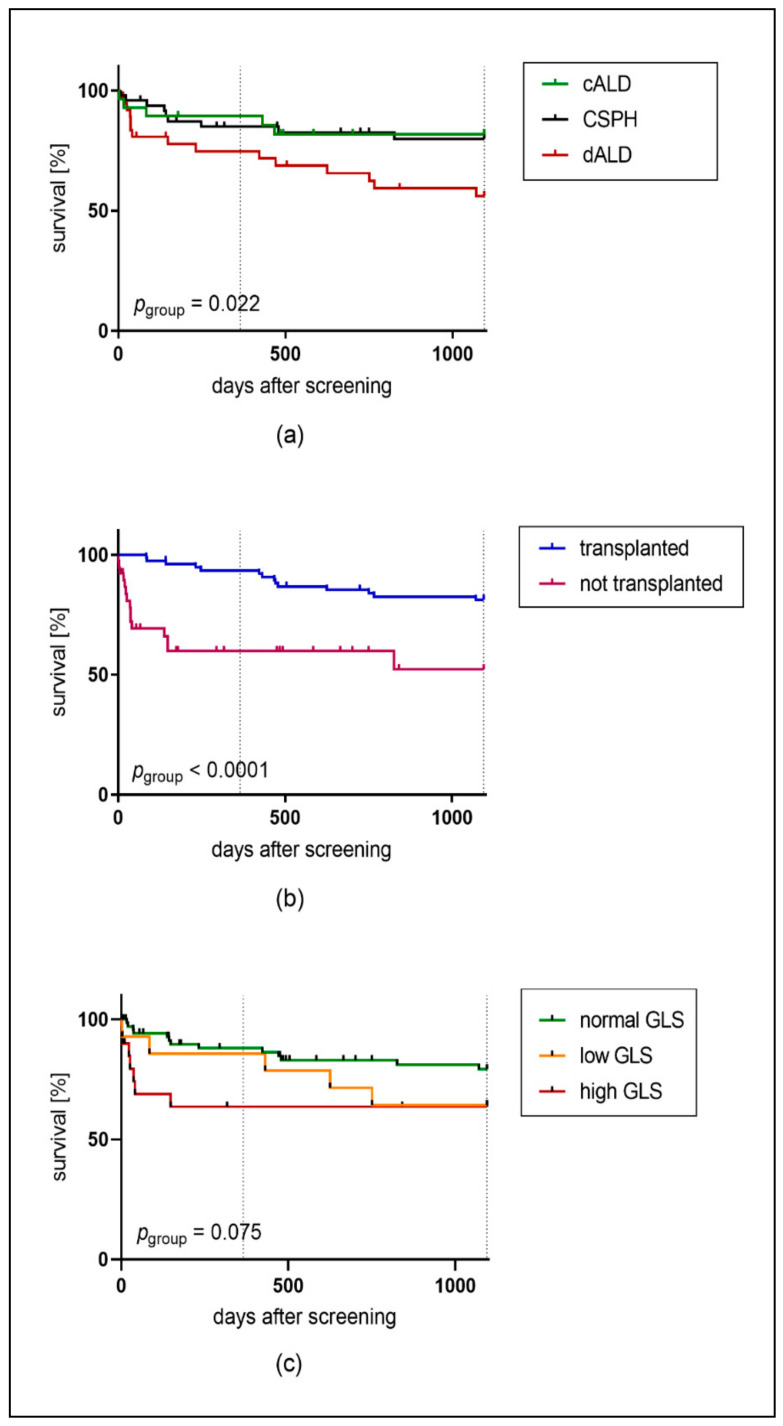
Kaplan-Meier survival curves: survival probability was displayed according to (**a**) liver disease groups, (**b**) liver transplantation and (**c**) global longitudinal strain (GLS) categories.

The Kaplan–Meier curves demonstrated that in the group with dALD, significantly fewer patients survived the 3 years of follow-up (56.1%) than those of the other groups (cALD: 81.8%; CSPH: 79.8%) (*p* = 0.022) (Figure 3a). In addition, non-transplant patients had significantly worse survival after an evaluation for liver transplantation than transplanted patients (non-transplant vs. transplant: one-year survival: 59,8% vs. 93.4%; three-year survival: 52.3% vs. 81.2%) (*p* < 0.001) (Figure 3b). Log-rank (Mantel–Cox) test based on the GLS categories revealed that patients with low and high GLS values tended to show a lower survival (64.3% and 61.9%) than patients with normal GLS (80.6%, *p* = 0.075) (Figure 3c).

Cox regression identified dALD (odds ratio (OR), 2.758; *p* = 0.048), MELD score, (OR, 1.145; *p* < 0.001), GLS category at rest (OR, 2.614; *p* = 0.033), body mass index (OR, 0.898; *p* = 0.041), and transplantation (OR = 0.277; *p* = 0.001) as predictors of mortality. After multivariate analysis, dALD (OR, 7.561; *p* = 0.045), MELD score (OR, 1.191, *p* = 0.001), low GLS at rest (OR, 16.482; *p* = 0.002), and transplantation (OR, 0.082; *p* = 0.001) kept a significant effect on mortality (Table 4).

The incidence of MACE differed significantly between the GLS categories (*p* = 0.019): patients with both low and high GLS values suffered more frequently from MACE than patients with normal GLS values (incidence with low GLS: 64%; normal GLS: 27%; high GLS: 43%).

## 4. Discussion

The results from this retrospective analysis of echocardiographic examinations in liver transplant candidates with normal ejection fraction identified low GLS values at rest as an independent predictor of mortality. Furthermore, survival was influenced by the severity of the underlying liver disease and by transplantation.

GLS is the preferred strain parameter for describing left ventricular systolic dysfunction in patients with heart failure and preserved ejection fraction (HFpEF). This is because the longitudinal subendocardial fibers are more sensitive to early changes, and therefore, the resulting functional alterations are better detected by GLS. Furthermore, GLS is independent of diastolic dysfunction [35]. A relevant variation in GLS can be observed despite a normal LVEF in many cardiac diseases. In contrast to LVEF, GLS demonstrated a significant correlation with invasive measurements of contractility and was less influenced by changes in afterload and preload [36].

This study indicates a similar relationship for liver transplant candidates: despite a normal LVEF, liver transplant candidates showed a strongly fluctuating GLS at rest. Most of our patients demonstrated normal values (67%), while 20% had high and 13% had low values, where low values indicate latent systolic dysfunction. Previous studies describe reduced [13,20,37] or normal GLS [38] at rest in patients with liver cirrhosis. In addition, advanced stages of liver disease led to an increase in EDVi and SVi. According to the results of Chowdhury et al. EDVi did not correlate with GLS [36]. Higher GLS values occurred more frequently in patients with CSPH and dALD. This would display the higher hemodynamic stress related to cirrhosis induced portal hypertension and might help to describe its severity.

In view of the interindividual differences in GLS, the question of relevance in terms of patient outcome arises. Particularly, as predicting the outcome is the most important issue, and possible parameters should be evaluated according to this criterion. There are fewer studies that examine the effect of systolic dysfunction on mortality in liver cirrhosis than those that look at diastolic dysfunction. Sampaio et al. calculated a hazard ratio of 1.67 (0.61–4.60; *p* = 0.322) if GLS was higher than −19.8% [39]. Recently, Turco et al. described the effect of hemodynamics on mortality in cirrhosis [40]: a cardiac output above 4.2 L/min or below 3.2 L/min was associated with increased mortality. Thus, hyperdynamic circulation related to decompensated cirrhosis as well as hypodynamic circulation related to CCM might increase mortality. This theory is reinforced by our data, as the results of this study indicate that the relationship between survival and GLS does not appear to be linear: both low and high GLS levels seem to be related to an increased risk of death. As high GLS values are associated with more advanced liver diseases, only low values were independent predictors of mortality. In contrast to high GLS values, low values were found in all groups. They seemed not to be associated with hemodynamic stress of portal hypertension and did not indicate CCM. It is unquestioned that the severity of cirrhosis affects survival [41], the underlying disease of the liver might therefore be the main reason for the observed increased mortality in patients with high GLS levels. In addition, it is known that liver transplantation improves survival, even in the presence of CCM [5]. This was also evident in our data. The cause of low GLS values remains speculative. Patient characteristics, except BMI, were not associated with low GLS values in the current investigation. Patients with low GLS values demonstrated higher BMI (30.4 ± 5.7 vs. 26.4 ± 5.0, *p* = 0.005). Lee et al. reported that obesity led to a decrease of GLS by 0.152% per 1 kg/m^2^ change in BMI [42].

Similar to survival, the correlation between the occurrence of MACE and GLS seemed not to be linear: both patients with high and low GLS were more likely to have MACE during the three years of follow-up, compared to patients with normal GLS.

In everyday clinical practice, DSE still plays a decisive role in screening for latent coronary artery disease in a pre-transplant setting [43]. Furthermore, the contractile reserve is often assessed by means of DSE [31]. For example, according to the criteria of the World Congress of Gastroenterology in 2005, DSE is used to diagnose systolic dysfunction and can thus support the diagnosis of cirrhotic cardiomyopathy [44]. However, it should be noted that these diagnostic criteria are increasingly being challenged, e.g., the criteria of the Cirrhotic Cardiomyopathy Consortium of 2019, suggest using strain analysis (GLS) at rest instead of DSE parameters [44].

In our study, the hemodynamic stress associated with high cardiac index and higher EDVi was, as expected, associated with an increase in contractility and thus GLS in most patients. The increase in GLS during DSE represents the contractile reserve. Contractile reserve is often used to assess myocardial function in progressive heart failure. In CCM, Moller et al. describe a reduced contractile reserve before function at rest is impaired [45]. At least for patients with non-ischemic cardiomyopathy with severely reduced LVEF, a prognostic benefit of a contractile reserve has been demonstrated: these patients have a prolonged survival and a reduced rate of cardiovascular events [46]. Nevertheless, the transferability to patients with end-stage liver disease is questionable: the results of our study show that patients had higher GLS and EDVi values at rest with increasing severity of liver disease, but all groups reached the same level during DSE. Since strain is determined by load and contractility, this means that both contractility and ventricular filling were equal at maximum stress. Accordingly, there seems to be a kind of maximum contractility, which obviously questions the relevance of the increase, since a high baseline inevitably leads to the expression of a false negative contractile reserve. The understanding and measurement of the contractile reserve should therefore be reconsidered at least in patients with end-stage liver disease. Nevertheless, a reduced contractile reserve, as a consequence of high values at rest and normal values at stress, will limit a further increase of cardiac output, which could be necessary during major surgery or severe illness.

### Limitations

As with most clinical strain data [19], the study was conducted retrospectively so some examinations, such as DSE, were not performed in every patient and some interesting parameters, such as brain natriuretic peptide, were not measured regularly. Furthermore, DSE does not induce exactly the same physiological response as physical stress. Nevertheless, an exercise stress test in patients with severe liver cirrhosis is often not possible due to their limited exercise capacity related to muscular weakness [47]. In addition, the echocardiographic examinations at rest and under stress were recorded with two different devices and the analysis was carried out with two different tools. However, it must be put into perspective that the results of the two analysis tools of GE EchoPac and TOMTEC Imaging Systems are known to show a very good correlation [48]. As with any echocardiographic studies using speckle tracking, the analysis is dependent on good image quality [49]. The exclusion of 11 patients from the strain analysis due to poor image quality may, for example, led to selection bias. In addition, the manual determination of the region of interest also creates the potential for error [19]. The reduced number of patients with DSE may also have influenced the results and the picture of the contractile reserve may have been different having a DSE in all patients. The unequal distribution of diagnoses in the different groups could also have been a source of bias, even though the diagnosis had no significant effect on left ventricular function as displayed by GLS or LVEF. Last but not least, the limited number of patients also limits the validity of the results.

## 5. Conclusions

GLS has been shown to be useful in predicting survival and MACE in patients with end-stage liver disease and normal LVEF: low and high GLS values were associated with increased mortality. Whereas low values are an independent predictor of mortality, high values are associated with advanced stages of liver disease, another independent predictor. Therefore, further cardiac evaluation should be considered in patients with low values, whereas high values would indicate a high hemodynamic stress level.

## Figures and Tables

**Table 1:** Patient characteristics (mean +/− SD).

	cALD (*n* = 29)	CSPH (*n* = 49)	dALD (*n* = 39)	P between Groups
Female (*n* (% of group))	12 (41%)	17 (35%)	11 (28%)	0.524
Age (years)	55.1 ± 7.3	57.0 ± 10.0	55.0 ± 10.1	0.538
BMI (kg/m^2^)	26.4 ± 5.1	26.9 ± 5.6	26.2 ± 4.0	0.830
Diagnosis cirrhosis (*n*)				<0.001
Acute liver failure	4 ^‡‡^	0	0	
C2 cirrhosis	3	16	18	
Viral cirrhosis	1	12	8	
PSC/PBC	6	2	1	
NASH cirrhosis	0	4	2	
Cryptic cirrhosis	0	6	7	
Tumor/cystic	10 ^‡‡^	5	0	
Other	5	4	3	
Decompensation (*n*)				
Hydropic	0 ^‡‡^	0 ^‡‡^	30 ^‡‡^	<0.001
TIPSS	0	0 ^‡^	10 ^‡‡^	<0.001
SBP	0	0 ^‡‡^	14 ^‡‡^	<0.001
HRS	0	0 ^‡‡^	15 ^‡‡^	<0.001
MELD score	12.9 ± 7.8	15.1 ± 6.8	18.3 ± 6.2 **	0.006
Creatinine (mg/dL)	1.7 ± 2.0	1.1 ± 0.6	1.6 ± 1.1	0.072
Bilirubin (mg/dL)	2.7 ± 4.7	5.4 ± 8.2	4.4 ± 5.1	0.214
INR	1.4 ± 1.7	1.4 ± 0.3	1.4 ± 0.5	0.905
Platelets (1/nL)	209 ± 80	92 ± 44 ***	88 ± 47 ***	<0.001
CRP (mg/L)	16.8 ± 20.6	10.1 ± 12.8	30.0 ± 50.7 ^†^	0.019
Sodium (mmol/L)	138 ± 2	137 ± 4	133 ± 7	0.063
Further blood analysis (g/dL)				
Hb	12.4 ± 2.1	11.6 ± 2.2	10.4 ± 2.3 ** ^†^	0.002
Albumin	3.5 ± 0.7	3.3 ± 0.7	2.9 ± 0.5 ** ^†^	0.002
Systolic BP (mmHg)	123 ± 17	116 ± 18	113 ± 17	0.106
Diastolic BP (mmHg)	75 ± 11	67 ± 15 *	67 ± 9 *	0.009
HR (1/min)	82 ± 13	71 ± 13 **	78 ± 16	0.005
Medication (*n* (% of group))				
Beta blocker	8 (28%) ^‡^	32 (65%)	21 (54%)	0.005
ACE inhibitor or AT_1_ antagonist	7	11	4	0.243
Diuretics	8 ^‡‡^	29	28	0.001
Other antihypertensive agents	4	3	1	0.186
Medical history (*n*)				
T1D	0	1	0	0.497
T2D	8	20	15	0.485
aHTN	8	22	11	0.165

* *p* ≤ 0.05, ** *p* ≤ 0.01, *** *p* ≤ 0.001 vs. cALD; ^†^ *p* < 0.05 vs. CSPH. ^‡^ < 0.008; ^‡‡^ < 0.001 vs. expected value. **Abbreviations:** ACE inhibitor, angiotensin-converting enzyme inhibitor; aHTN, arterial hypertension; AT_1_ antagonist, angiotensin II receptor subtype 1 antagonist; BMI, body mass index; BP, blood pressure; cALD, compensated advanced liver disease; CRP, C-reactive protein; CSPH, compensated advanced liver disease with clinically significant portal hypertension; dALD, decompensated advanced liver disease; Hb, hemoglobin; HR, heart rate; HRS, hepatorenal syndrome; Hydropic, hydropic decompensation; INR, international normalized ratio; MELD, model of end-stage liver disease; other antihypertensive agents: drugs such as calcium channel blocker; alpha2-agonists, dihydralazine, endothelin receptor antagonists, phosphodiesterase type 5 inhibitors; SBP, spontaneous bacterial peritonitis; T1D, type 1 diabetes mellitus; T2D; type 2 diabetes mellitus; TIPSS, transjugular intrahepatic portosystemic shunt.

**Table 2:** Echocardiographic parameters at rest (mean +/− SD).

	cALD (*n* = 29)	CSPH (*n* = 49)	dALD (*n* = 39)	*P*_group_
Dimensions				
LVEDD (mm)	44.3 ± 4.5	46.6 ± 4.6	47.0 ± 5.3	0.057
LVESD (mm)	26.1 ± 4.5	27.4 ± 5.4	28.6 ± 7.3	0.221
Septum ED (mm)	10.5 ± 1.9	9.7 ± 2.1	9.7 ± 1.8	0.144
Systolic parameters				
LVEF (%)	61.1 ± 5.9	62.9 ± 4.8	63.3 ± 4.4	0.185
TAPSE (mm)	23.5 ± 4.2	26.3 ± 5.4	27.1 ± 5.9 *	0.017
GCS (%)	−25.5 ± 5.0	−27.0 ± 5.1	−27.5 ± 5.8	0.354
GRS (%)	28.6 ± 10.3	29.7 ± 14.4	25.0 ± 10.0	0.227
GLS at rest (%)	−18.8 ± 2.4	−21.9 ± 3.2 ***	−22.4 ± 3.6 ***	<0.001
LV twist (°)]	7.7 ± 9.1	7.4 ± 9.0	7.5 ± 9.1	0.980
Diastolic parameters				
E/A	0.95 ± 0.32	1.24 ± 0.44 **	1.11 ± 0.27	0.004
DT (ms)	228 ± 52	223 ± 54	214 ± 58	0.586
E (m/s)	0.60 ± 0.15	0.73 ± 0.17 **	0.77 ± 0.19 ***	<0.001
E’ (m/s)	0.069 ± 0.022	0.073 ± 0.024	0.071 ± 0.017	0.668
E/E’	10.0 ± 3.5	11.2 ± 4.2	11.1 ± 3.2	0.369
TR velocity (m/s)	1.4 ± 0.3	1.3 ± 0.2	1.4 ± 0.2	0.414
LA vol index (mL/m^2^)	19.2 ± 6.3	25.9 ± 6.7 **	26.8 ± 9.0 ***	0.001
Diast dysf (*n*)				0.021
I°	13	8	6	
II°	3	5	3	
III°	0	5	1	

* *p* ≤ 0.05, ** *p* ≤ 0.01, *** *p* ≤ 0.001 vs. cALD. **Abbreviations:** cALD, compensated advanced liver disease; CSPH, compensated advanced liver disease with clinically significant portal hypertension; dALD, decompensated advanced liver disease; Diast dysf, diastolic dysfunction; DT, deceleration time; E, mitral valve early diastolic velocity; E’, mitral annulus early diastolic velocity; ED, end diastolic; E/A, ratio of early transmitral flow velocity to late diastolic filling velocity caused by atrial contraction; E/E’, ratio of the mitral valve early diastolic flow velocity to the mitral annulus early diastolic flow velocity; GCS, global circumferential strain; GLS, global longitudinal strain; GRS, global radial strain; LA vol index, maximum left atrial volume index; LV twist, left ventricular twist according to [32]; LVEDD, left ventricular end-diastolic diameter; LVESD, left ventricular end-systolic diameter; LVEF, left ventricular ejection fraction; TAPSE, tricuspid annular plane systolic excursion; TR velocity, tricuspid regurgitation peak velocity.

**Table 3:** Stress echo parameters (mean +/− SD).

	cALD (*n* = 8)	CSPH (*n* = 26)	dALD (*n* = 18)	*P*_group_
MAP (mmHg)				0.311
Baseline	96 ± 14	80 ± 14	84 ± 9	
Peak	93 ± 33	92 ± 21	97 ± 22	
HR (1/min)				<0.004 ^†††^
Baseline	84 ± 9	67 ± 13 **	83 ± 18 ^‡‡^	
Peak	151 ± 9	143 ± 14	139 ± 7	
CI (L/min/m^2^)				0.371 ^†††^
Baseline	2.5 ± 0.8	2.5 ± 0.6	2.8 ± 0.8	
Peak	3.3 ± 0.9	3.8 ± 1.0	3.8 ± 1.4	
GLS (%)				0.546 ^†††^
Baseline	−20.3 ± 3.6	−21.8 ± 2.8	−22.5 ± 3.9	
Peak	−24.2 ± 4.2	−24.1 ± 4.2	−24.4 ± 4.6	
LVESVi (mL/m^2^)				0.319 ^†††^
Baseline	16.5 ± 4.6	22.4 ± 5.8	19.6 ± 5.2	
Peak	11.4 ± 6.1	11.7 ± 4.8	12.9 ± 5.5	
LVEDVi (mL/m^2^)				0.047 ^†††^
Baseline	46.1 ± 11.4	58.8 ± 10.6 *	56.0 ± 13.2	
Peak	33.4 ± 10.5	38.0 ± 10.2	40.6 ± 14.5	
SWi (mmHg·mL/m^2^)				0.673 ^††^
Baseline	2875 ± 903	2912 ± 740	2824 ± 518	
Peak	2037 ± 845	2420 ± 883	2655 ± 1259	

^††^*p* < 0.01, ^†††^ ≤ 0.001 time within groups; * *p* < 0.05, ** *p* ≤ 0.01 vs. cALD; ^‡‡^ *p* ≤ 0.01 vs. CSPH. **Abbreviations:** cALD, compensated advanced liver disease; CI, cardiac index; CSPH, compensated advanced liver disease with clinically significant portal hypertension; dALD, decompensated advanced liver disease; GLS, global longitudinal strain; HR, heart rate; LVEDVi, left ventricular end-diastolic volume index; LVESVi, left ventricular end-systolic volume index; MAP, mean arterial pressure; SWi, stroke work index.

**Table 4:** Parameters that could predict mortality. Univariate and multivariate Cox regression analysis displaying odds ratios (ORs) und *p*-values.

	Univariate		Multivariate	
	OR	*p*-Value	OR	*p*-Value
Group				
CSPH	1.068	0.906	2.728	0.332
dALD	2.758	0.048	7.561	0.045
MELD score	1.145	<0.001	1.191	0.001
GLS category at rest				
GLS low	1.871	0.234	16.483	0.002
GLS high	2.614	0.033	1.128	0.864
GLS peak	1.061	0.410		
Delta GLS < 2%	1.460	0.573		
LVEF peak	0.933	0.120		
Delta LVEF < 5%	2.070	0.260		
BMI	0.898	0.041	0.898	0.086
Transplantation	0.277	0.001	0.082	0.001
Age	1.005	0.817		
Diastolic dysfunction				
I°	1.144	0.750		
II°	0.588	0.475		
III°	0.675	0.702		

**Abbreviations:** At rest, during resting echocardiography (beta-blocker not paused); BMI, body mass index; CSPH, clinically significant portal hypertension; dALD, decompensated advanced liver disease; delta, difference between the values at rest and at maximum dobutamine rate; DSE, dobutamine stress echocardiography; GLS, global longitudinal strain; GLS category, three categories of GLS values (low, normal and high) based on the thresholds published by Asch et al. [30]; group, three groups based on the severity of liver cirrhosis according to the classification of de Franchis et al. [25]; high, GLS < −26 (female) and < −24 (male); low, GLS > −18 (female) and > −17 (male); LVEF, left ventricular ejection fraction; MELD, model of end-stage liver disease; OR, odds ratio; peak, point in time during DSE with maximum dobutamine rate; transplantation, transplantation during the 3 years of follow-up.
